# The relationship between the age of onset of hypertension and chronic kidney disease: a cross-sectional study of the American population

**DOI:** 10.3389/fcvm.2024.1426953

**Published:** 2024-12-12

**Authors:** Lanlan Qiu, Bo Wu

**Affiliations:** ^1^ Department of Cardiology, Longyan People’s Hospital, Longyan, Fujian, China; ^2^ Department of Cardiology, Longyan First Affiliated Hospital of Fujian Medical University, Longyan, Fujian, China

**Keywords:** hypertension, onset age, early-onset hypertension, CKD, NHANES

## Abstract

**Background:**

Hypertension can damage multiple target organs. The younger the age of onset of hypertension is, the greater the risk of cardiovascular disease (CVD) and cardiovascular death. Chronic kidney disease (CKD) is a complication of hypertension, but few studies have investigated the relationship between the age of onset of hypertension and CKD.

**Objective:**

We investigated the relationship between the age of onset of hypertension and CKD.

**Method:**

We analyzed data from the National Health and Nutrition Examination Survey (NHANES) 2007–2018. A total of 30,613 participants were assigned to one of four groups. Group 1, no hypertension (*n* = 19,516); Group 2, age of onset <35 years (*n* = 2,180); Group 3, 35≤ age of onset <45 years (*n* = 2,128); and Group 4, age of onset ≥45 years (*n* = 6,789). Logistic regression analysis was used to evaluate the relationship between the age of onset of hypertension and CKD.

**Results:**

After adjusting for potential confounders, a younger age at onset of hypertension was associated with a greater risk of developing CKD compared with the absence of hypertension (Group 2 OR: 2.52, 95% CI: 1.53–4.14, *P* < 0.001; Group 3 OR: 1.59, 95% CI: 1.01–2.51, *P* = 0.048; Group 4 OR: 1.54, 95% CI: 1.00–2.38, *P* = 0.050).

**Conclusions:**

There was a strong association between the age of onset of hypertension and CKD. The younger the age of onset of hypertension is, the greater the risk of CKD.

## Introduction

Hypertension can damage the heart, brain, kidney, and other target organs (1), and it has a heavy global disease burden (2). Notably, epidemiological findings show that the hypertension population is becoming younger and that the proportion of patients with early-onset hypertension is increasing (3). Compared with patients with late-onset hypertension, those with early-onset hypertension appear to be at greater risk for a poor prognosis.

Available evidence suggests that patients with early-onset hypertension are at increased risk for ventricular remodeling and coronary artery calcification (4). In addition, patients with a younger age of onset of hypertension had a greater risk of nonfatal cardiovascular disease (CVD) events and CVD mortality than patients with an older age of onset (5, 6). However, most studies on the age of onset of hypertension and the risk of poor prognosis are limited to CVD, and the dangers of early-onset hypertension are far from well understood (7). The relationship between the age of onset of hypertension and other clinical outcomes needs further study (8).

CKD is a common complication of hypertension (9). Approximately 20% of hypertensive patients progress to CKD each year (10). The prevalence of CKD has been increasing over the past 20 years, with one of the causes being the increasing prevalence of hypertension (11). The main cause of death in CKD patients is usually not CKD itself but CVD complications due to CKD (12). Recently, the American Heart Association (AHA) proposed the concept of cardiovascular–kidney–metabolic (CKM) syndrome (13). In the United States, nearly 60% of adults are in CKM stage 2 (additional metabolic risk factors or moderate- or high-risk CKD) or higher (13). This further confirms the strong association between CKD and CVD incidence. Therefore, CKD cannot be overlooked when exploring the harms of early-onset hypertension.

Exploring the influence of early-onset hypertension on CKD will be beneficial for the clinical management of cardiorenal diseases. Our aim was to explore the relationship between the age of onset of hypertension and CKD and to provide references for identifying hypertensive patients with a high risk of CKD.

## Methods

### Study population

**Figure 1:**
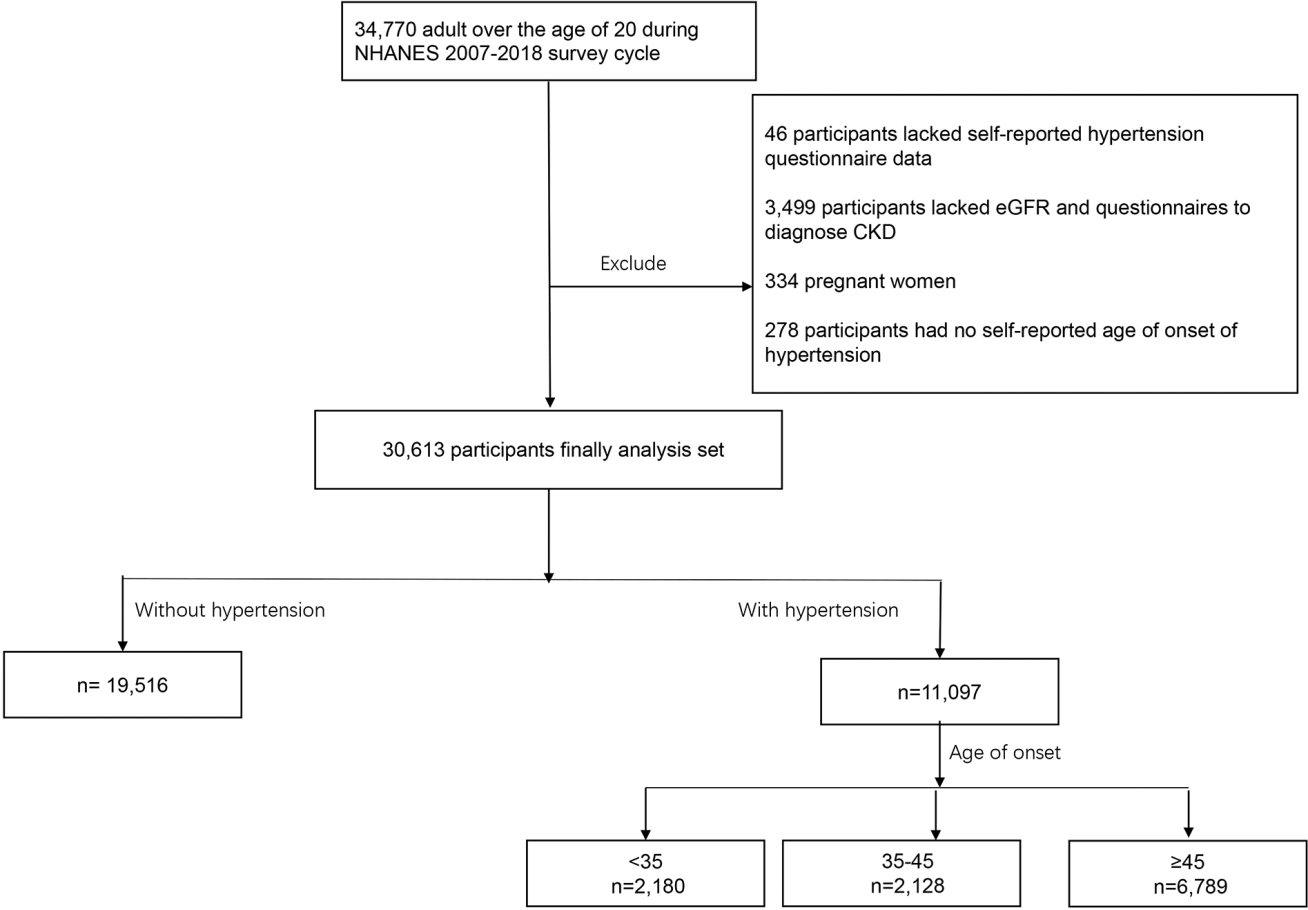
Flowchart of the study design.

The National Health and Nutrition Examination Survey (NHANES) includes data on the health and nutritional status of adults and children in the United States beginning in the early 1960s. It is an annual self-report survey of different populations and health topics in a nationally representative sample of approximately 5,000 people located in counties throughout the country. Data on the age of onset of hypertension were available from participants from 2007 to 2018. In the NHANES 2007–2018, 34,770 participants were over the age of 20 years. A total of 46 patients lacked hypertension data, 278 lacked information on the age of onset, and 3,499 lacked the estimated glomerular filtration rate (eGFR) data, randomized urinary albumin/creatinine ratio (UACR) data, or data needed to diagnose CKD. Additionally, 334 pregnant women were excluded. The remaining 30,613 people were included in the study (Figure 1).

### Definitions, primary outcomes, and patient characteristics

Participants who answered “yes” to the NHANES questionnaire item that asked “Has a doctor ever told you that you have hypertension?” were considered hypertensive. The age of onset was also self-reported as the answer to the question “How old were you when your doctor first told you that you had high blood pressure?” In addition, the duration of exposure to hypertension was obtained by subtracting the age of onset from the current age.

The primary outcome was CKD. Participants with an eGFR of <60 ml/min/1.73 m^2^ and/or a UACR of >30 mg/g were considered to have CKD (14).

The participant characteristics that were variables of interest were age, sex, ethnicity, body mass index (BMI), smoking status, education level, poverty income ratio (PIR), smoking status, and alcohol use. The diagnosis of comorbidities was assigned in the case of an affirmative response to the question “Has a doctor or other health professional ever told you that you had diabetes mellitus (DM), CVD including coronary heart disease, congestive heart failure, heart attack, stroke, or angina?” Systolic blood pressure (SBP) was the mean pressure measured at the mobile examination center and at home via a mercury sphygmomanometer. Laboratory measurements, such as creatinine (Cr), triglyceride (TG) and total cholesterol (TC) levels, were assayed with automated hematological analysis equipment. eGFR was determined via the CKD-EPI-SCr 2009 equation (15). The detailed procedures for obtaining laboratory measurements are described on the website of the National Center for Health Statistics (https://wwwn.cdc.gov/Nchs/Nhanes/).

### Population grouping method

Participants without hypertension composed the reference group (Group 1). Hypertensive patients were divided into three groups according to their self-reported age of onset: <35 years (Group 2), <35 years and <45 years (Group 3), and ≥45 years (Group 4). The grouping method was the same as that used in previous studies (16).

### Statistical analysis

The weights recommended by the NHANES were used for complex sampling analysis for all the statistical analyses. To reflect the representativeness of the sample, continuous variables are reported as the means (standard errors), and categorical variables are reported as numbers (percentages). Baseline characteristics were compared via analysis of variance or *t*-tests for continuous variables and chi-square tests for categorical variables. We used logistic regression analysis to evaluate the association between the age of onset of hypertension and CKD. Both estimates and probabilities were based on values recommended by the NHANES. Model 1 was adjusted for age. Model 2 was adjusted for age, sex, and ethnicity. Model 3 was adjusted for all potential confounders. To reduce retrospective bias because of current age, we stratified the data by current age. We also stratified patients by sex and the presence of DM. To investigate the possibility of a nonlinear relationship between age at the onset of hypertension and CKD, a regression cubic spline (RCS) analysis was performed.

The statistical analysis was performed via the survey package in R statistics (version 4.2.2; R Foundation for Statistical Computing, Vienna, Austria). Two-sided *p*-values <0.05 indicated significance for all analyses.

## Results

### Participant characteristics

There were 30,613 participants, 11,097 with hypertension and 19,516 without hypertension. Those with hypertension were older, had higher lipid levels, included higher proportions of smokers and drinkers, were more likely to have CVD and CKD, and had lower levels of education and income compared with those without hypertension (Table 1). Hypertensive patients were grouped by age of onset. Group 2 (age of onset <35 years) included 2,180 participants, Group 3 (age of onset ≥35 years and <45 years) included 2,128 participants, and Group 4 (age of onset ≥45 years) included 6,789 participants. Patients with older-onset hypertension were currently older and more likely to be female. Participants with an older age at hypertension onset had higher levels of education and income and were less likely to drink. In addition, the older the age of onset of hypertension is, the greater the prevalence of DM and CVD. There were no significant differences in blood lipid levels among the age groups. The results are shown in Table 2.

**Table 1:** Baseline characteristics of the study population (weighted).

	Non-HTN (*n* = 19,516)	HTN (*n* = 11,097)	*P*-value
Age, mean (SE), years	43.0 (0.2)	57.7 (0.2)	<0.001
Male, *n* (%)	9,643 (48.9)	5,390 (48.7)	0.716
Ethnicity, *n* (%)			<0.001
Mexican American	3,353 (10.0)	1,318 (5.7)	
Non-Hispanic Black	3,407 (9.4)	2,921 (13.5)	
Non-Hispanic White	7,884 (65.5)	4,726 (69.1)	
Other Hispanic	2,169 (6.5)	1,062 (4.7)	
Other	2,703 (8.6)	1,070 (7.0)	
Education level			<0.001
<High school	4,499 (14.7)	3,013 (17.9)	
High school	4,266 (22.0)	2,701 (25.0)	
Collage or above	1,0735 (63.3)	5,370 (57.0)	
PIR	3.0 (0.0)	2.9 (0.0)	0.012
SBP, mean (SE), mmHg	117.8 (0.2)	130.9 (0.3)	<0.001
BMI, mean (SE), kg/m^2^	28.0 (0.1)	31.4 (0.1)	<0.001
TG, mean (SE), mg/dl	114.70 (1.28)	142.27 (2.41)	<0.001
TC, mean (SE), mg/dl	193.11 (0.52)	193.13 (0.71)	0.984
LDL-C, mean (SE), mg/dl	114.59 (0.50)	111.91 (0.66)	<0.001
Smoking, *n* (%)	8,045 (41.6)	5,507 (50.3)	<0.001
Alcohol^a^, *n* (%)	12,770 (71.3)	6,157 (63.3)	<0.001
DM, *n* (%)	2,112 (7.8)	3,845 (28.8)	<0.001
Duration of hypertension, *n* (%)			<0.001
<1 years	19,516 (100.0)	597 (5.6)	
1–4.9 years	0 (0.0)	2,698 (25.3)	
5–9.9 years	0 (0.0)	2,264 (22.3)	
≥10 years	0 (0.0)	5,538 (46.8)	
CVD, *n* (%)	885 (3.8)	2,513 (19.6)	<0.001
CKD, *n* (%)	2,129 (8.9)	3,681 (27.9)	<0.001

PIR, poverty income ratio; SBP, systolic blood pressure; BMI, body mass index; TG, triglyceride; TC, total cholesterol; LDL-C, low-density lipoprotein cholesterol; DM, diabetes mellitus; CVD, cardiovascular disease; CKD, chronic kidney disease.

^a^ The average number of drinks consumed per day over the past 12 months.

**Table 2:** Baseline characteristics of participants at different ages of onset (weighted).

	<35 (*n* = 2,180)	35–45 (*n* = 2,128)	≥45 (*n* = 6,789)	*P*-value
Age, mean (SE), years	42.5 (0.5)	51.6 (0.3)	65.7 (0.2)	<0.001
Male, *n* (%)	1,081 (54.9)	1,072 (52.8)	3,237 (44.8)	<0.001
Ethnicity, *n* (%)				<0.001
Mexican American	228 (7.4)	242 (6.3)	848 (4.8)	
Non-Hispanic Black	716 (18.4)	665 (16.4)	1,540 (10.6)	
Non-Hispanic White	837 (61.6)	810 (64.8)	3,079 (73.5)	
Other Hispanic	189 (5.4)	198 (5.2)	675 (4.2)	
Other	210 (7.2)	213 (7.2)	647 (6.8)	
Education levels, *n* (%)				0.004
<High school	463 (15.8)	519 (16.9)	2,031 (19.1)	
High school	564 (24.9)	497 (22.9)	1,640 (25.9)	
Collage or above	1,152 (59.3)	1,112 (60.2)	3,106 (55.0)	
PIR	2.7 (0.1)	3.1 (0.1)	3.0 (0.0)	<0.001
SBP, mean (SE), mmHg	128.6 (0.4)	129.5 (0.6)	132.4 (0.4)	<0.001
BMI, mean (SE), kg/m^2^	32.8 (0.2)	32.4 (0.2)	30.4 (0.1)	<0.001
TG, mean (SE), mg/dl	148.58 (4.80)	143.99 (3.70)	139.24 (3.03)	0.185
TC, mean (SE), mg/dl	191.36 (1.16)	195.19 (1.54)	193.05 (0.79)	0.104
LDL-C, mean (SE), mg/dl	111.58 (1.55)	114.38 (1.47)	111.08 (0.87)	0.153
Smoking, *n* (%)	1,076 (49.2)	1,005 (48.6)	3,426 (51.3)	0.275
Alcohol^a^, *n* (%)	1,378 (70.2)	1,248 (65.3)	3,531 (60.0)	<0.001
DM, *n* (%)	570 (20.3)	726 (28.9)	2,549 (32.1)	<0.001
Duration of hypertension, *n* (%)				<0.001
<1 years	76 (3.8)	80 (3.7)	441 (7.0)	
1–4.9 years	419 (21.7)	509 (25.7)	1,770 (26.6)	
5–9.9 years	359 (19.1)	365 (20.6)	1,540 (24.1)	
≥10 years	1,326 (55.4)	1,174 (50.0)	3,038 (42.4)	
CVD, *n* (%)	384 (13.8)	422 (16.5)	1,707 (22.9)	<0.001
CKD, *n* (%)	561 (21.1)	556 (20.9)	2,564 (33.0)	<0.001

PIR, poverty income ratio; SBP, systolic blood pressure; BMI, body mass index; TG, triglyceride; TC, total cholesterol; LDL-C, low-density lipoprotein cholesterol; DM, diabetes mellitus; CVD, cardiovascular disease; CKD, chronic kidney disease.

^a^ The average number of drinks consumed per day over the past 12 months.

### Relationship between the age of hypertension onset and CKD

There were 2,129 CKD patients (8.9%) without hypertension and 3,681 (27.9%) with hypertension. In models adjusted only for current age, a younger age at onset of hypertension was associated with a greater risk of developing CKD compared with the absence of hypertension (Group 2 OR: 3.17, 95% CI: 2.70–3.72, *p* < 0.001; Group 3 OR: 2.08, 95% CI: 1.79–2.42, *p* < 0.001; and Group 4: OR: 1.91, 95% CI: 1.75–2.08, *p* < 0.001). After full adjustment for potential confounders (age, sex, race/ethnicity, education, PIR, smoking, alcohol use, BMI, TG, TC, LDL-C, DM, CVD, duration of exposure to hypertension), the association between age of onset of hypertension and CKD remained significant (Group 2 OR: 2.52, 95% CI: 1.53–4.14, *P* < 0.001; Group 3 OR: 1.59, 95% CI: 1.01–2.51, *P* = 0.048; Group 4 OR: 1.54, 95% CI: 1.00–2.38, *P* = 0.050) (Table 3).

**Table 3:** The association between the age of onset of hypertension and CKD (weighted).

	Model 1		Model 2		Model 3	
	OR (95% CI)	*P*-value	OR (95% CI)	*P*-value	OR (95% CI)	*P*-value
Non-HTN	Ref		Ref		Ref	
Onset age						
<35 years	3.17 (2.70,3.72)	<0.001	3.13 (2.66,3.67)	<0.001	2.52 (1.53,4.14)	<0.001
≥35 and <45 years	2.08 (1.79,2.42)	<0.001	2.05 (1.76,2.38)	<0.001	1.59 (1.01,2.51)	0.048
≥45 years	1.91 (1.75,2.08)	<0.001	1.87 (1.72,2.05)	<0.001	1.54 (1.00,2.38)	0.050

Model 1: Adjusted by age.

Model 2: Adjusted by age, sex, and race/ethnicity.

Model 3: Adjusted for age, sex, race/ethnicity, education, PIR, smoking status, alcohol status, BMI, TG, TC, LDL-C, duration of hypertension, DM, and CVD.

### Subgroup analysis

**Figure 2:**
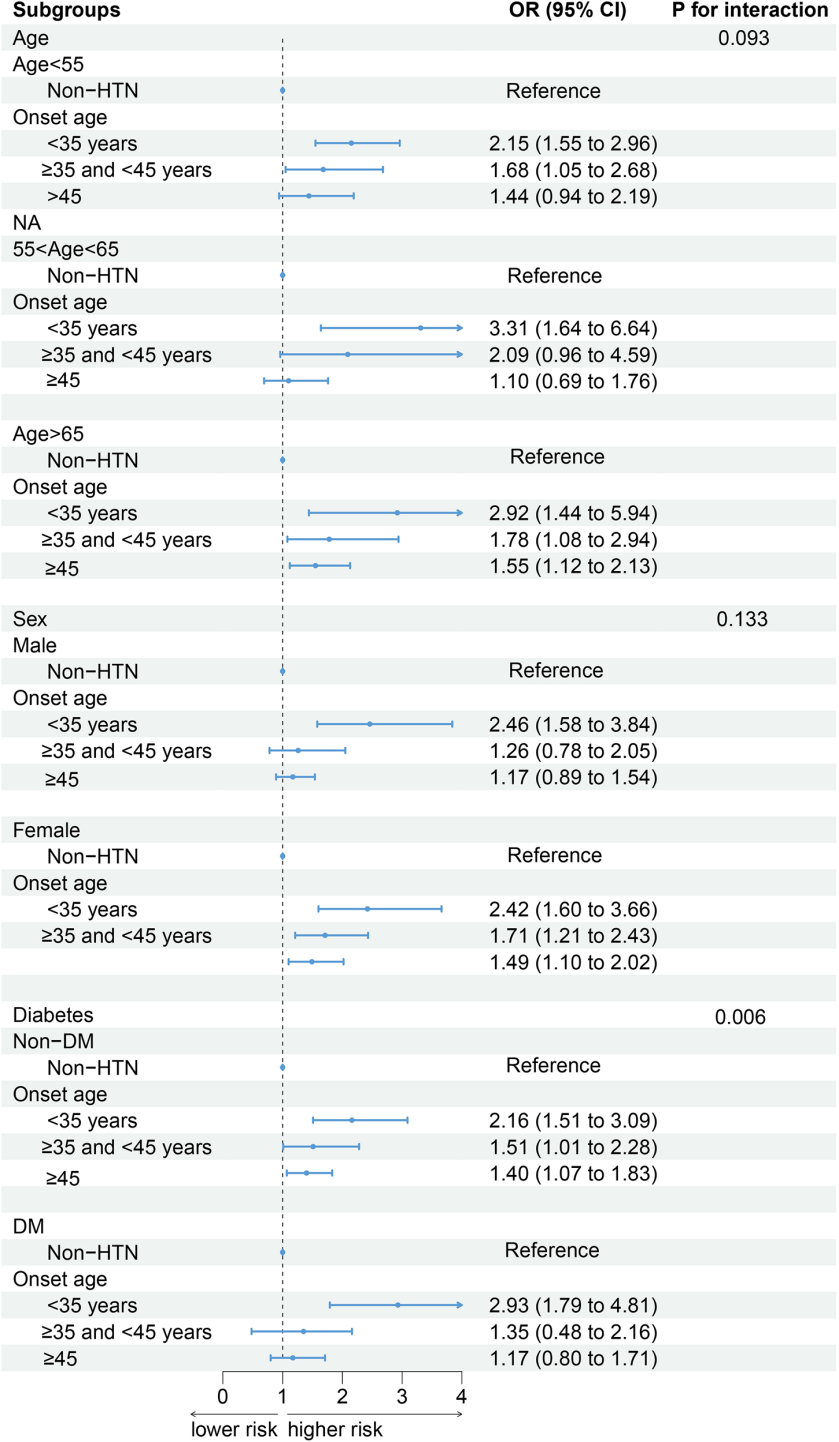
The associations between the age of onset of hypertension and the incidence of CKD in different subgroups (weighted). Adjusted for age, sex, race/ethnicity, education, PIR, smoking, alcohol use, BMI, TG, TC, LDL-C, DM, CVD, duration of exposure to hypertension.

After the participants were stratified by current age, and sex status, the association between the age of onset of hypertension and CKD did not change. Compared with patients without hypertension, those with a younger age of hypertension onset have a greater risk of developing CKD (Figure 2). In the analysis stratified by diabetes status, an interaction was observed between DM and the age of onset for hypertension. Among patients with DM, Although the risk of CKD was lower in the group with the later onset of hypertension, this was not statistically significant. For those with an onset age of <35 years: OR = 2.93 (95% CI: 1.79–4.81); For those with an onset age between 35 and 45 years: OR = 1.35 (95% CI: 0.48–2.16); For those with an onset age of ≥45 years: OR = 1.17 (95% CI: 0.80–1.71).

### Regression cubic splines

**Figure 3:**
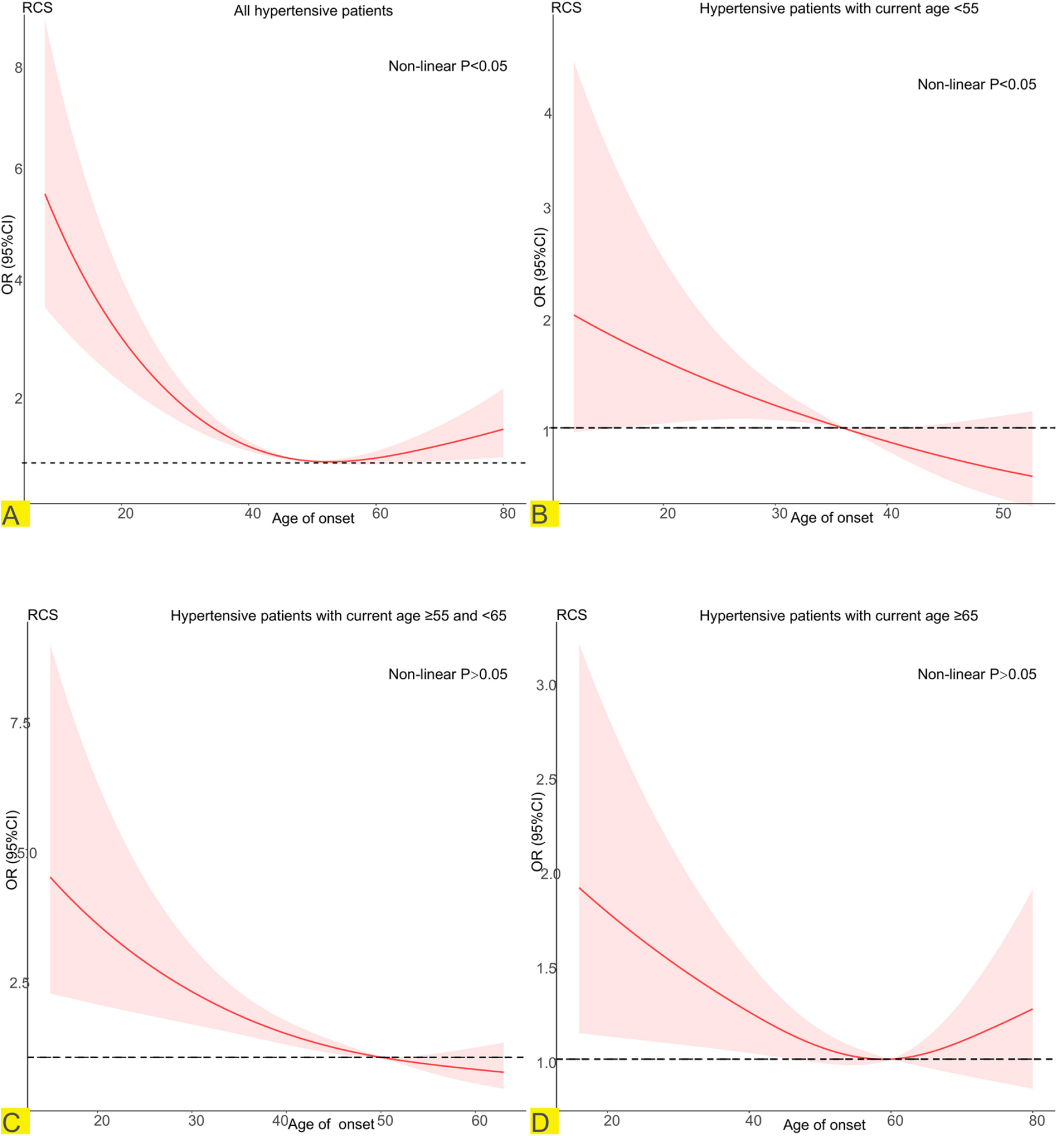
Potential nonlinear relationship between age at onset of hypertension and CKD (weighted). Adjusted for age, sex, race/ethnicity, education, PIR, smoking, alcohol use, BMI, TG, TC, LDL-C, DM, CVD, duration of exposure to hypertension. **(A)** Relationship between age of onset of hypertension and CKD in all hypertensive patients. **(B)** Relationship between age of onset of hypertension and CKD in hypertensive patients with current age <55. **(C)** Relationship between age of onset of hypertension and CKD in hypertensive patients with current age ≥55 and <65. **(D)** Relationship between age of onset of hypertension and CKD in hypertensive patients with current age ≥55.

In hypertension patients, RCS revealed a U-shaped relationship between the age of onset of hypertension and CKD (Figure 3A). The risk of developing CKD in participants who developed hypertension before 49 years of age decreased with increasing age of onset. In participants who became hypertensive after age 49, the risk of developing CKD increased with age of onset. When stratified according to the current age of the hypertensive patients. In hypertensive patients younger than 65 years of age (Figures 3B,C), the earlier the onset of hypertension is, the greater the risk of CKD. When the current age of hypertensive patients was greater than 65 years (Figure 3D), there was a U-shaped association between the age of hypertension onset and the risk of CKD.

## Discussion

This large cross-sectional study revealed that the age at onset of hypertension was strongly associated with the risk of developing CKD. The younger the age of onset is, the greater the risk of CKD. The relationship between age at onset of hypertension and CKD did not change after stratification by current age and sex.

Many studies have investigated the relationship between the age of onset of hypertension and poor prognosis in hypertensive patients. They reported that a younger age of onset was associated with an increased risk of ventricular remodeling and coronary artery calcification compared with those without hypertension (4). In addition, a lower age of onset of hypertension was found to be a risk factor for nonfatal cardiovascular events, including myocardial infarction, ischemic stroke, and hemorrhagic stroke; cardiovascular death; and all-cause death (5, 6, 17). These findings indicate that the age at hypertension onset is a non-negligible factor in the stratification of hypertension prognosis. However, the age at onset is not included in risk assessment in existing hypertension guidelines (18, 19). A review by Suvila et al. noted that the complications of hypertension are not limited to cardiovascular disease and death and that more evidence is needed to include age at onset of hypertension in the assessment of risk and to translate it into clinical practice (8).

CKD is a major complication of hypertension (20), and its increasing prevalence worldwide (21) adds to the global medical economic burden. In the United States alone, the annual cost of treating CKD increased from $28 billion 10 years ago to $50 billion at the time of writing (22, 23). Current evidence shows that the increase in the prevalence of hypertension is an important reason for the increase in the prevalence of CKD (11). Consequently, it is necessary to extend the study of the age of onset of hypertension to CKD. In our study, the results revealed that patients who developed hypertension at a younger age had a greater risk of developing CKD than did those who developed hypertension at an older age. After stratification by sex and DM, the relationship between age at onset of hypertension and CKD did not change. Notably, patients who developed hypertension later in life were actually older. As this was a retrospective study, it was difficult to avoid. To minimize bias, patients were stratified by age into groups of <55 years and 55–65 years of age for subgroup analysis, the results of which were consistent with the main results. The younger the age of onset of hypertension is, the greater the risk of CKD. Although a U-shaped association was found in people ≥65 years of age, the 95% CI was not statistically significant.

In exploring the possibility of a nonlinear relationship between age at onset of hypertension and CKD, the RCS indicated a U-shaped relationship between age at onset of hypertension and CKD. However, the relationship disappeared after the patients were stratified by their current age. As described above, patients with late-onset hypertension in this retrospective analysis were also older. Current age, as a risk factor for CKD (24, 25), may mask the linear relationship between the age of onset of hypertension and CKD. After controlling for the effect of current age in a stratified analysis, a younger age of onset remained associated with a greater risk of developing CKD. Therefore, our results are still reliable.

Additionally, it is necessary to note that the duration of exposure to hypertension is also an important factor for the occurrence of CKD in patients with hypertension. Therefore, the negative correlation between the age of onset of hypertension and CKD may be because patients who develop hypertension later in life have a shorter exposure time to high blood pressure. However, in Model 3 of this study, the exposure time to hypertension was also included in the regression model for analysis. The results still hold statistical significance. This finding indicates that the negative correlation between the age of onset of hypertension and CKD is independent of the duration of exposure to hypertension.

In the stratification based on DM status, it was observed that among diabetic patients, only those with hypertension onset at age <35 years have a higher risk of developing CKD. For individuals who have both DM and hypertension, the primary cause of CKD may stem from DM rather than hypertension. The speed at which DM patients progress to CKD is often more rapid than that of hypertension patients (26, 27). Therefore, the significance of the onset age of hypertension may be overshadowed by DM.

However, the reason why patients with early-onset hypertension are more likely to develop CKD may not be due only to substandard treatment for hypertension. Several studies have suggested that there is a genetic risk of early-onset hypertension; people whose parents have early-onset hypertension are more than three times as likely to develop high blood pressure as those whose parents do not (17). There is evidence that the M235T angiotensinogen gene variant is associated with familial early-onset hypertension (28). Numerous studies have reported a strong correlation between M235T and CKD (29–31). In addition, Yoshiji et al. reported that the MOB3C-TMOD4, COL6A3 and COL6A5 gene loci are associated with the occurrence of early-onset hypertension and CKD (32). We suspect that patients with early-onset hypertension may be genetically more likely to develop CKD than those with late-onset hypertension. Given that these findings cannot be verified by a retrospective study, prospective studies are needed to verify this hypothesis.

In this cross-sectional study, the sample size was large enough to be powered to represent 252,462,687 people in the United States, making our results more reliable. Our study revealed that the age of onset of hypertension was associated not only with cardiovascular disease risk, all-cause mortality, and cardiovascular death in hypertensive patients but also with CKD in hypertensive patients. This finding fills a gap in age-related studies on hypertension and provides more evidence for stratifying the prognosis of hypertensive patients. Although current hypertension management guidelines do not include age of onset when assessing the prognosis of hypertensive patients, considering the correlation between early-onset hypertension and CKD as well as CVD, incorporating age of onset into clinical guidelines could enhance risk stratification. This would enable health care providers to more effectively identify high-risk patients. For example, younger patients with early-onset hypertension may benefit from more aggressive monitoring and management strategies to reduce the risk of CKD and other complications. Therefore, early identification of the harms associated with early-onset hypertension and its integration into clinical practice for the treatment and management of hypertensive patients is highly important.

## Limitations

There were several study limitations. First, it was subject to the limitations inherent in a retrospective analysis. The relationship between the age of onset of hypertension and CKD could be interpreted only as a correlation rather than as a causal relationship. Second, whether hypertension was the cause of a diagnosis of CKD could not be determined. Third, the age of onset was self-reported by patients, which may be different from the actual age of hypertension. However, previous studies have demonstrated consistency among self-reported age groups with hypertension as defined by objective age groups, and our results are still reliable (16).

## Conclusions

Our results indicated a strong association between age at onset of hypertension and CKD. The younger the age of onset of hypertension is, the greater the risk of CKD. For patients with early-onset hypertension, early intervention should be prioritized.

## Data Availability

The raw data supporting the conclusions of this article will be made available by the authors, without undue reservation.
